# Data Resource: Vascular Risk in Adult New Zealanders (VARIANZ) datasets

**DOI:** 10.23889/ijpds.v4i1.1107

**Published:** 2019-09-02

**Authors:** S Mehta, R Jackson, DJ Exeter, BP Wu, S Wells, AJ Kerr

**Affiliations:** 1 Section of Epidemiology and Biostatistics, School of Population Health, University of Auckland, Auckland, New Zealand; 2 Middlemore Hospital, Auckland, New Zealand

**Keywords:** Cardiovascular disease, administrative data, record linkage

## Abstract

**Introduction:**

The Vascular Risk in Adult New Zealanders (VARIANZ) datasets contain a range of routinely-collected New Zealand health data relevant to cardiovascular disease (CVD) and related conditions. The datasets enable exploration of cardiovascular-related treatment, service utilisation, outcomes and prognosis.

**Processes:**

Each dataset is constructed by anonymised individual-level linkage of eight national administrative health databases to identify all New Zealand adults aged ≥20 years who have recorded contact with publicly-funded New Zealand health services during a given year from 2006 onwards, when data quality is considered sufficient.

**Data contents:**

Individual-level data for each VARIANZ dataset can include variables covering demography, dispensing of cardiovascular disease (CVD) preventive medications and prior hospitalisations for atherosclerotic CVD, heart failure, atrial fibrillation and diabetes. If required, VARIANZ datasets can be individually linked to follow-up national routinely collected health data in subsequent years, including all-cause mortality events and fatal/non-fatal CVD events, to create VARIANZ longitudinal cohorts. Bespoke linkage can also be undertaken to include other national and regional administrative health data such as non-CVD related hospitalisations in order to explore CVD comorbidities or novel risk factors. Furthermore, a subset of the VARIANZ datasets based on specific health contacts (such as CVD hospitalisations only) can also be identified, and some data can be requested for years prior to 2006. The New Zealand routinely-collected health databases used to construct the VARIANZ datasets do not capture primary care diagnostic classifications or certain CVD risk factor data such as smoking status, blood pressure or lipid profiles.

**Conclusion:**

The Vascular Risk in Adult New Zealanders (VARIANZ) datasets capture the majority of the New Zealand population in a given year and are available from 2006 onwards, or earlier than 2006 for some datasets based on specific health contacts. VARIANZ data can be used to explore a range of research questions regarding management, outcomes and prognosis for CVD.

## Highlights

- The Vascular Risk in Adult New Zealanders (VARIANZ) datasets enable exploration of cardiovascular-related treatment, outcomes and prognosis.
- Data from eight national administrative health databases are anonymously linked at the individual-level to identify New Zealanders aged 20 years or older who interacted with publicly-funded health services during a specified year (from 2006 onwards) and were still alive at the end of the year. For example, the VARIANZ 2006 dataset includes 2 543 577 New Zealand residents alive on 31/12/2006 and captures 85% of the 2006 New Zealand Census Estimated Resident population aged 20 years or older.
- Baseline VARIANZ datasets focussed on specific health contacts, such as CVD hospitalisations only, can also be constructed and some data can be requested for years prior to 2006.
- VARIANZ datasets for a given year can be individually linked to data in subsequent years (for example, all-cause mortality events, fatal and non-fatal CVD events and CVD medication dispensing) to create VARIANZ cohort studies.
- VARIANZ datasets can contain a range of variables for an individual, customised to the study question being answered, including demography, cardiovascular disease (CVD) pharmacotherapy, and prior hospitalisations for atherosclerotic CVD, atrial fibrillation, heart failure and diabetes. Other relevant New Zealand administrative health data can also be added as required to explore CVD comorbidities or novel risk factors.

## Background

Internationally, there is growing interest in the use of linked health data to explore population-level trends in social and public health issues [1]. In New Zealand, administrative data regarding a wide range of publicly-funded health interactions are routinely collected across the country. Assignment of a unique National Health Index (NHI) identifier by the New Zealand Ministry of Health to every person who utilises the New Zealand health system enables data across different health databases to be linked at the individual level.

### Rationale for the Vascular Risk in Adult New Zealanders (VARIANZ) datasets

The Vascular Risk in Adult New Zealanders (VARIANZ) datasets are currently being used for analyses conducted as part of the Vascular Informatics using Epidemiology and the Web (VIEW) research programme based at the University of Auckland. The VARIANZ dataset for 2006 was initially constructed and linked to five years of follow-up administrative health data to develop policy-focussed five-year cardiovascular disease (CVD) risk prediction equations that could be applied across the country, and to enable validation of these new equations in a range of New Zealand population groups. However, since constructing the VARIANZ 2006 dataset, various research questions have arisen related to the treatment, outcomes and prognosis of CVD and related conditions. Therefore, the VIEW team has developed the capacity to construct similar datasets for successive years and to identify subsets of the full datasets based on specific health contacts. Furthermore, the baseline datasets can be linked to health data from subsequent years to create longitudinal cohort study data.

### New Zealand public health system

New Zealand has a population of around 4.5 million people. Around three quarters of public health funding is allocated by the New Zealand Ministry of Health to the 20 District Health Boards that purchase and provide public health services in their area, including public hospitals. Through Primary Health Organisations (PHOs)[2], DHBs also fund primary health care services that are provided by General Practices or other providers belonging to that PHO[3]. Patients can choose to enrol with a PHO, which entitles that individual to access various benefits including cheaper General Practitioner services[4]. Re-enrolment is required every three years for patients to continue receiving funded care by a particular PHO, and 93% of all New Zealanders were PHO-enrolled as of July 2018[4]. Patients eligible for publicly-funded healthcare in New Zealand who do not enrol with a PHO can still access primary care services but will not receive the additional benefits of belonging to a PHO such as a reduced service co-payment.

The remaining quarter of public health funding is used by the New Zealand Ministry of Health to fund a variety of nationally-administered services such as screening programmes[2]. The New Zealand Ministry of Health collects and administers databases containing routine administrative data related to interactions with public health services in New Zealand, and the VIEW team obtains annual extracts from selected health databases. Around one third of the population have some form of private health insurance, but the private health sector accounts for less than 20% of all health spending in New Zealand[5] and less than 5% of all CVD-related acute hospital care.

## Processes

### Overview

The full VARIANZ datasets can be constructed from 2006 onwards, with CVD-related history variables and, if required, follow-up variables able to be customised to the study question being answered. While it is possible to create VARIANZ populations from data recorded in 2005 or earlier, we have selected 2006 as the cut-off for generating full VARIANZ datasets largely because of poorer pharmaceutical dispensing data quality prior to this time. Reliable matching of dispensing episodes to NHI numbers has improved over time; 27% in 2002, 64% in 2004, 91% in 2006 and 96% or higher from 2011 onwards. (Analytical Services, New Zealand Ministry of Health, personal communication 2014) Consequently, medications may have been dispensed to some individuals but were not recorded against that person’s NHI identifier. Furthermore, a census was held in New Zealand in 2006, and therefore the population capture among demographic subgroups was able to be precisely determined for the 2006 VARIANZ dataset.

The VARIANZ datasets include all adults aged 20 years or older in contact with the New Zealand publicly-funded health system during a given year who were recorded in at least one of eight[6-13] national health datasets obtained from the New Zealand Ministry of Health. For example, the VARIANZ 2006 dataset includes baseline demographic variables, prior CVD or cardiac-related hospitalisations and current preventive CVD pharmacotherapy among people who interacted with the health care system during the period 1 January 2006 and 31 December 2006.

Nevertheless, some study questions may not require the full VARIANZ baseline datasets. VARIANZ datasets based on specific health contacts, such as CVD hospitalisations only, can therefore also be constructed, and non-pharmaceutical-dispensing data can be requested for years prior to 2006 if available. (Table 1 lists the start dates of availability for each source dataset).

A VARIANZ (baseline) dataset can then be linked to subsequent (follow-up) administrative health data for a selected period of interest to create a VARIANZ cohort study. The 2006 VARIANZ cohort study, for instance, comprises baseline data from the VARIANZ 2006 dataset and follow-up data regarding CVD medications dispensed and fatal and non-fatal CVD events occurring nationally between 1 January 2007 and 31 December 2011.

**Figure 1: Overview of the process to construct VARIANZ data resources available for research:**
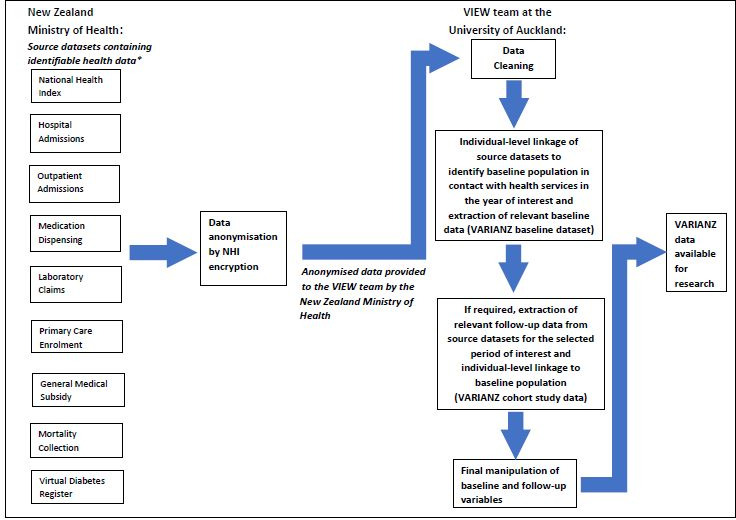
*The first eight source datasets are used to identify the full baseline VARIANZ population for a given time period. The Virtual Diabetes Register is used to determine diabetes status at baseline (or during follow-up if required), but does not identify any additional individuals at baseline as it draws on selected data from five of the other datasets. The Virtual Diabetes Register is, therefore, not included in the linkage process to identify the full baseline population. Subsets of the full VARIANZ baseline datasets based on specific health contacts can be identified from the relevant source datasets, and linked to follow-up data if required.

Figure 1 summarises the process of converting the raw administrative data into VARIANZ data that are ready for research. Further information about source datasets is provided in Table 1.

### Source and linkage of datasets

#### Source Data

The VARIANZ datasets comprise anonymised data sourced from the routinely collected New Zealand administrative health databases[6-14] listed in Table 1. All source data are subject to quality checks by the Ministry of Health prior to being made available to the VIEW research team. (An overview of key quality checks undertaken by the Ministry of Health is provided in Appendix 1). Post-delivery profiling of each annual data extract is also carried out by the VIEW team to check data consistency, including analyses of volumes and frequency over time across key variables (such as those listed in the ‘Data Contents’ section) in each dataset. Further information regarding the quality checks undertaken for specific datasets can be obtained on request from the VIEW research programme.

**Table 1: Source datasets for Vascular Risk in Adult New Zealanders (VARIANZ) data:** 

Dataset	Type of Data contained (start date)
National Health Index	Encrypted National Health Index number, date of birth and demographic data. (Data are available for all individuals with an NHI number)
Hospital admissions	Publicly-funded hospitalisations: date of admission, date of discharge and ICD-coded discharge diagnoses. (1988-)
Outpatient admissions	Publicly-funded outpatient and community visits (2006-)
Medication dispensing	Government-subsidised medication dispensing claims from community pharmacies (2006-)
Laboratory Claims	Publicly-funded laboratory tests requested; name of test recorded but does not include test results (2003-)
Primary Health Organisation (PHO) Enrolment	Enrolment data from primary health care provider organisations. Individuals must re-enrol every 3 years to continue receiving funded care by a particular PHO (2004-)
General Medical Subsidy	Funding claims by Primary Care professionals for patient visits; captures patients not enrolled with a regular primary care provider (2002-)
Mortality Collection	Date of death and ICD-coded cause of death (1988-)
Virtual Diabetes Register	Diabetes diagnosis as at the date of interest (e.g. 31 December 2006 for VARIANZ 2006 data) based on hospitalisations, outpatient visits, retinal screening, dispensing of diabetes-specific medications and laboratory HbA1c testing. (2005-)

#### NHI Unique Personal Identifiers, Data Encryption and Linkage

A NHI unique alphanumeric identifier is assigned to every person who has contact with the New Zealand health system, and is available for more than 98% of the national population[15]. Encryption of NHI numbers enables anonymisation of health data and is undertaken by replacing the seven actual characters with 64 other alphanumeric characters. The process of NHI encryption for the VARIANZ data is undertaken by the New Zealand Ministry of Health prior to source data being made available to the VIEW research programme.

Some individuals may inadvertently be issued more than one NHI personal identifier if existing NHI details are not available when attending a health service or provider for the first time. The Ministry of Health identifies individuals with multiple NHI identifiers using probabilistic matching, followed by manual record review of any newly detected potentially matching NHIs, of which approximately two thirds are eventually identified as separate individuals. A 2012 Ministry audit estimates that the probabilistic matching process misses 0.2% of all individuals with multiple NHIs, but health services and providers can alert the Ministry to investigate potential duplicate personal identifiers, which triggers a clerical review. The accuracy of the manual record review process has not yet been formally evaluated (Identity Team, National Digital Services, New Zealand Ministry of Health, personal communication 2019).

The Ministry of Health annually provides the VIEW team with an updated list of all ‘primary’ encrypted NHI numbers in use and any associated ‘secondary’ encrypted NHI numbers. People with multiple NHIs are identified by the VIEW team using this list (e.g. n=540 876 for individuals in the VARIANZ 2006 dataset aged 20 years or older) and their health records are subsequently reassigned to the single encrypted ‘primary’ NHI number designated by the Ministry of Health. Anonymised individual-level linkage of the relevant national health databases is then carried out using the encrypted NHI identifiers. The core datasets required to identify the baseline population in contact with health services in a given year are simultaneously linked. Further linkage is then undertaken to construct baseline variables and/or follow-up variables. As the linkage process is deterministic and involves unique personal identifiers, the linkage rate across source datasets once multiple NHIs have been reassigned to a single ‘primary’ NHI is 100%.

#### Sampling Strategy

The VARIANZ datasets include all adults aged 20 years or older who have any of the following interactions with publicly-funded health services in a given year:

- New or re-enrolments and/or a health service contact with a Primary Health Organisation
- any inpatient or outpatient secondary care service contact
- dispensing of any Government-subsidised medication by a community pharmacy
- any publicly-funded community laboratory test claim

For the PHO enrolment component of inclusion criterion “a”, the VARIANZ datasets capture those individuals who choose to enrol for the first time or re-enrol with a PHO during the year of interest. As previously mentioned, individuals remain enrolled with a given PHO for three years, at which point they have the option of re-enrolling. As emigration details are not accessible in the New Zealand health data, the tight criterion regarding PHO enrolment is used as a default for the VARIANZ sampling strategy to minimise inclusion of individuals who had been enrolled with a PHO in the years immediately preceding the baseline year of interest but who had actually moved overseas by 31 December of that year. However, the population capture of the VARIANZ datasets can be increased by including all individuals who voluntarily enrolled for the first time or re-enrolled with a PHO during the preceding three years, although this will inevitably also capture some individuals who have moved overseas by the baseline date of interest. (Please note that although more than 98% of the New Zealand population have been assigned an NHI personal identifier indicating previous contact with the New Zealand health system, only 93% of individuals were enrolled with a PHO in 2018 with even lower enrolment rates in 2006).

As previously mentioned, subsets of the full VARIANZ datasets can also be identified from specific health contacts, such as CVD hospitalisations only.

### Ethical Approval

The Vascular Informatics using Epidemiology & the Web (VIEW) research programme was approved by the Northern Region Ethics Committee Y in 2003 (AKY/03/12/314) and by the national Multi-Region Ethics Committee in 2007 (MEC/01/19/EXP). Ethical approval was also granted in 2011 for mapping the geographic variation of CVD across New Zealand (MEC/11/EXP/078). Subsequent ethics re-approvals have been obtained annually. Individual patient consent is not required for inclusion in the dataset as all data are anonymised.

## Data Contents

### Variables

The 2006 VARIANZ cohort data comprised over 200 individual-level variables and Table 2 presents an overview of the included measures, which provide an indication of the variables that can be included in the VARIANZ data resources. However, the VARIANZ datasets and cohort studies can incorporate any relevant routine administrative New Zealand health data, enabling the history variables and, if required, follow-up variables to be customised to the CVD-related study question being answered.

We identified people with a history of hospital admissions for CVD (coronary heart disease, haemorrhagic and ischaemic cerebrovascular events including transient ischaemic attacks, peripheral vascular disease and CVD-related procedures), heart failure and atrial fibrillation between 1 January 1993 and 31 December 2006. The Virtual Diabetes Registry for 2006, administered by the New Zealand Ministry of Health, was used to categorise individuals with diabetes as at 31 December 2006. This dataset contains every individual identified as having diabetes based on a series of criteria (listed in Table 1) applied to national routinely collected health data[14]. CVD event variables identified deaths and first hospitalisations for coronary heart disease, haemorrhagic and ischaemic cerebrovascular events (including transient ischaemic attacks), heart failure and peripheral vascular disease between 1 January 2007 and 31 December 2011. The list of ICD 9 and 10 codes corresponding to CVD history and events and the methodology used to construct the Virtual Diabetes Registry are both available on request from the VIEW research programme.

Socioeconomic status was assessed using the New Zealand Deprivation Index (NZDep). NZDep is derived for small areas using nine variables from New Zealand Census data related to eight dimensions of socioeconomic deprivation[16]. The NZDep score was assigned to individuals according to their area of residence and is available for all VARIANZ datasets. The New Zealand Index of Multiple Deprivation is a newer small area-based measure of socioeconomic status sourced from routine administrative data that enables disaggregation of the seven component domains of deprivation, and is available for VARIANZ datasets from 2010 onwards[17].

The default format for incorporating medication dispensing in the VARIANZ datasets is as binary variables capturing dispensing at least once in six-month intervals over the time period of interest. However, medication dispensing data are available at a level of detail that would enable other metrics such as medicine possession ratios or proportion of days covered to be incorporated into the datasets. Other formats for medication dispensing variables can, therefore, be requested by researchers.

**Table 2: Overview of the Vascular Risk of Adult New Zealanders 2006 (VARIANZ 2006) cohort study data:** * Baseline medication dispensing was recorded in binary variables capturing dispensing at least once between 1 July 2006 and 31 December 2006 ^#^ Medication dispensing was recorded in binary variables capturing dispensing at least once in sequential six-month periods between 1 January 2007 and 31 December 2011. Ł Loop diuretic dispensing at baseline can be used as an indicator of heart failure, including among individuals who have not previously been hospitalised with the condition.

Time Period	Type of Data	Variables
Baseline	General	Encrypted unique identifier
		Date of birth
		Dates of last health contact (last primary care contact and last health contact of any type in 2006)
		Cause of death ICD codes

	Demographic	Age
		Sex
		Ethnic group
		Socioeconomic deprivation status
		New Zealand resident

	Geographic	Small area measures based on address
		Primary health care organisation
		District Health Board

	History of CVD and cardiac-related hospitalisations: 1 Jan 1993 - 31 Dec 2006	CVD (binary variable and date of first hospitalisation)
		Heart failure (binary variable and date of first hospitalisation)
		Atrial fibrillation (binary variable and date of first hospitalisation)
		Diabetes – binary variable and dates for first occurrence of relevant health contacts (outpatient visit etc)

	CVD risk for individuals without history of CVD or heart failure hospitalisations	Five-year risk of fatal or non-fatal CVD

	Medication dispensing:* 1 Jul 2006 - 31 Dec 2006	Blood-pressure lowering medications
		Lipid-lowering medications
		Antiplatelet and anticoagulant medications
		Loop-diuretics^Ł^

1 Jan 2007 - 31 Dec 2011	Mortality-general	First CVD-related hospitalisation (binary variable and date of first hospitalisation)
		First CVD-related hospitalisation by sub-type of CVD (binary variables)
		CVD-related death (binary variable and date of death)
		First CVD event –death or hospitalisation (binary variable and date of event)

	Medication dispensing^#^	Blood-pressure lowering medications
		Lipid-lowering medications
		Antiplatelet and anticoagulant medications

### Population Capture

#### VARIANZ 2006 Dataset

The methodology employed captured 2 575 786 people for the VARIANZ 2006 dataset. After exclusion of those who had a health contact in 2006 but died before the end of that year (26 910 individuals) and non-New Zealand residents (5 299 men and women), 2 543 577 people remained. A comparison of this latter group to the 2006 New Zealand Census Estimated Resident population is shown in Table 3. The VARIANZ 2006 dataset captured 85% of the 2006 New Zealand Census Estimated Resident population aged 20 years or over, with increasing percentages identified among people aged 55 years and older.

**Table 3: Demographic characteristics of the Vascular Risk in Adult New Zealanders 2006 (VARIANZ 2006) baseline population compared to the 2006 New Zealand Census Estimated Resident (ER) population:** *Total excludes 26 910 people who had a health contact in 2006 but died before 31 December 2006 and 5 299 people who were not New Zealand residents at 31 December 2006. ^#^Male and female totals exclude 139 people whose sex could not be identified in the national datasets ^The 2006 VARIANZ study population identified 454 more individuals aged 85 years or older than the 2006 New Zealand Census Estimated Resident population, potentially due to the high proportion of this age group who utilise health services coupled with underestimation of the Census ER population aged 85 years and over as a result of census undercount. ^Ł^2006 New Zealand Census ER population figures for Maori, Pacific, Asian and Other groups are sourced from the 2006 New Zealand Estimated Resident Population supplied by Stats New Zealand. Estimates for Chinese and Indian groups were not available from Stats New Zealand and were subsequently sourced from the 2006 New Zealand Estimated Resident Population that was constructed by Counties Manukau District Health Board (CMDHB) in Auckland, New Zealand. If an individual identified with more than one ethnic group, CMDHB assigned a single prioritised ethnic group using the following prioritisation order: Maori; Pacific, South-East Asian, Chinese, Indian, Other Asian, Other. Due to rounding, the CMDHB 2006 New Zealand Census Estimated Resident Population aged 20 years or older comprised 2 982 335 individuals.

Demographic	VARIANZ 2006 baseline population n (%)	2006 New Zealand Census ER population n (%)	Difference between VARIANZ 2006 and 2006 Census ER populations n (% of Census ER)
Total	2 543 577*	2 982 740	439 163 (15%)
Sex:			
Male	1 140 283^#^ (45%)	1 433 980 (48%)	293 697 (20%)
Female	1 403 155^#^ (55%)	1 548 760 (52%)	145 605 ( 9%)
Age:			
20-34 years	652 170 (26%)	837 560 (28%)	185 390 (22%)
35-44 years	521 108 (20%)	635 050 (21%)	113 942 (18%)
45-54 years	486 247 (19%)	568 810 (19%)	82 563 (15%)
55-64 years	389 470 (15%)	429 670 (14%)	40 200 ( 9%)
65-74 years	263 268 (10%)	275 700 ( 9%)	12 432 ( 5%)
75-84 years	172 720 ( 7%)	177 780 ( 6%)	5 060 ( 3%)
85 years and over	58 594 ( 2%)	58 140 ( 2%)	-454 (0%)^
Ethnicity:Ł			
Maori	260 871 (10%)	343 050 (12%)	82 765 (24%)
Pacific	121 642 ( 5%)	159 520 ( 5%)	37 878 (24%)
Asian	165 689 ( 7%)	284 960 (10%)	119 271 (42%)
Chinese	56 325 ( 2%)	121 110 ( 4%)	65 738 (54%)
Indian	60 616 ( 2%)	80 609 ( 3%)	19 993 (25%)
Other	1 995 377 (79%)	2 195 210 (74%)	199 833 ( 9%)

The lower capture of younger individuals in the VARIANZ 2006 dataset is likely to be related to better average health, with reduced associated utilisation of health services, than among older individuals. Furthermore, younger individuals are more likely than older New Zealand residents to travel overseas in the short or medium term. New Zealand residents who were overseas during 2006 could still be counted in the New Zealand 2006 Census population as responses can be provided from overseas, but these individuals would not appear in the New Zealand routinely collected health data unless they accessed New Zealand health services during the year.

Non-Indian Asian peoples, in particular, as well as the high-risk Maori, Pacific and Indian communities are under-represented in the VARIANZ 2006 dataset compared to the 2006 Census Estimated Resident population, and the reasons are likely to be multifactorial. One contributing issue is potential differences in recorded ethnicity for individuals between routine health data and the Census-based Estimated Resident Population. Ethnicity is self-reported in New Zealand for both sources, and responses may change over time or in different settings[18, 19]. Lack of familiarity by recent immigrants with the structure of the New Zealand system may also lead to reduced health system interaction, including under-enrolment with PHOs. A proportion of recent Asian immigrants, particularly those of Chinese ethnicity, may also seek health care in other countries, as a consequence of spending significant periods of time overseas or preferentially returning to their country of origin for medical care[20]. The reduced capture of Maori and Pacific peoples in the VARIANZ datasets also likely reflects both the younger age structure of these ethnic groups, and variable healthcare access by ethnicity in New Zealand[21].

The differential capture within VARIANZ datasets by age and ethnic group as compared to the Census-based Estimated Resident Population is relatively stable over time and should be explicitly considered in all analyses. The analytical methodology employed to address this issue will depend on the study question but, where possible, age-specific and ethnic-specific results ought to be presented. Although CVD-related incidence and prevalence estimates for younger age groups will be inflated (since younger individuals with CVD-related health contacts will be disproportionately captured in the VARIANZ data), CVD also occurs most infrequently among the younger age bands so absolute rates are likely to still be relatively low. Similarly, exaggeration of CVD-related estimates is unlikely to be meaningful among Chinese individuals as this sub-population is at low risk for CVD. However, overestimation of hospitalisations, mortality and medication dispensing among high-risk Maori, Pacific and Indian groups requires more careful consideration, especially for Maori and Pacific peoples where inequities in unmet need exist.

#### Population Capture from 2006 onwards

Table 4 presents the number of individuals aged 20 years and older identified in the VARIANZ datasets for each year from 2006 to 2016 inclusive as compared to national Estimated Resident population estimates released annually by Statistics New Zealand. The population capture increased between 2006 and 2014, likely due to improved primary care enrolment numbers across the country. However, the estimated population capture fluctuates from year to year. The increasing proportion of the Estimated Resident population that are not captured by the VARIANZ datasets in 2015 and 2016 is likely to be influenced by increasing numbers of immigrants, particularly from Asian countries, among whom (as already noted) reduced utilisation of publicly-funded New Zealand health services is observed.

**Table 4: Vascular Risk in Adult New Zealanders (VARIANZ) baseline population aged 20 years or older compared to the New Zealand Estimated Resident (ER) population estimates* for 2006-2016 inclusive:** *ER population estimates at a given time after the most recent Census are derived by adjusting Census night population figures for undercounting including residents who were overseas, as well as adjustments for births, deaths and net migration. Of the years listed, New Zealand censuses were undertaken in 2006 and 2013.

Year	VARIANZ baseline population	ER Population	Difference n (% of ER population)
2006	2 543 577	2 982 740	439 163 (14.7%)
2007	2 603 163	3 016 220	413 057 (13.7%)
2008	2 654 867	3 047 270	392 403 (12.9%)
2009	2 712 406	3 084 480	372 074 (12.1%)
2010	2 765 473	3 125 470	359 997 (11.5%)
2011	2 812 263	3 158 750	346 487 (11.0%)
2012	2 844 106	3 185 460	341 354 (10.7%)
2013	2 888 404	3 220 820	332 416 10.3%)
2014	2 945 120	3 285 020	339 900 (10.3%)
2015	2 999 693	3 364 600	364 907 (10.8%)
2016	3 009 749	3 453 220	443 471 (12.8%)

## Example Uses

### Changes in CVD primary preventive pharmacotherapy over five years

The VARIANZ 2006 cohort study data have been used to examine changes in CVD preventive pharmacotherapy over five years among the national cohort aged 30-74 years without a prior history of CVD, in preparation for developing new five-year risk prediction models. This study demonstrated that one in five of the 1 766 584 individuals included in the study population received baseline treatment and that only modest treatment changes occurred over five years. In terms of risk prediction, therefore, baseline use of CVD medications is an important consideration when estimating CVD risk and will capture the majority of the effect of treatment in five-year models[22].

### Development and validation of policy-focussed five-year CVD risk equations

Five-year risk equations were subsequently derived and validated using the 2006 VARIANZ cohort data with the intention of informing policy formulation and strategic health planning for CVD prevention among people without a previous history of CVD. These equations are the first models that enable CVD risk to be estimated at the individual-level across an entire country and can be applied to the populations identified from VARIANZ datasets. The equations have multiple potential applications, including identification of high-risk sub-populations or regions where more intensive risk management strategies are warranted. This ‘precision health policy’ approach to CVD prevention will complement current efforts at the individual clinical level using clinical risk tools[23].

### CVD profile of older New Zealanders

A VARIANZ dataset comprising 514 710 people aged 65 years or older was constructed for 2010 to determine disease prevalence and CVD risk factors among this age group, with follow-up data identified from 2011-2015 inclusive to enable estimation of five-year event rates. This analysis provides the most comprehensive and contemporary overview of CVD among the elderly in New Zealand. The prevalence of CVD across the cohort in 2010 was found to be 31%, and a sizeable proportion of those without a prior history of CVD at baseline remained event-free during the following five years. In particular, the time-to-event similarities between the 65-74 years and 75-84 year group provide preliminary evidence that CVD risk management recommendations, which currently encompass those aged up to 74 years, should be extended to include individuals up to 84 years of age[24].

### Statin maintenance in the three years following acute coronary syndrome

Linked national hospitalisation and mortality data were used to construct a subset VARIANZ dataset comprising 11 348 individuals aged 35-84 years who were discharged from New Zealand hospitals with acute coronary syndrome (ACS) and were alive 90 days post discharge. This dataset was linked to pharmaceutical dispensing data to examine patterns of statin use. In the three-year period following ACS, one third of individuals overall were not adequately maintained on statins, but 82% of those adequately treated with a statin in the first year also had a medicine possession ratio of ≥80% during the third year of follow-up. Younger individuals and those of Maori and Pacific ethnicity were less likely to be maintained on long-term statin therapy. These results identify target sub-populations for improved statin use and suggest that if adequate statin maintenance can be achieved in the first year post-ACS, then therapy is likely to be subsequently maintained[25].

## Current Projects and International Context

### Current projects

Risk prediction models focussed on the elderly are currently being derived and validated using VARIANZ cohort data. A comorbidity index for individuals with chronic obstructive pulmonary disease, for which CVD is one of the most common comorbid conditions, is also being developed by Danish collaborators from the University of Southern Denmark using a VARIANZ cohort comprising individuals hospitalised with COPD.

### Opportunity for international comparisons

To our knowledge, there are no CVD-specific data resources with comprehensive population capture available for other countries. Nevertheless, there are a number of regional and national projects that provide opportunities for international comparisons. CVD-related UK analyses could be undertaken using the cardiovascular disease research using linked bespoke studies and electronic health records (CALIBER) data resource that links electronic health records from a subset of consenting UK primary care practices to a clinical registry for acute coronary syndromes and other UK data collections [26]. The Secure Anonymised Information Linkage Databank that provides whole-population linked data for Wales and the Kent Integrated Dataset[27] that links health data for all residents of Kent and Medway also enable UK comparisons. Similar datasets could be constructed from the Western Australia Data Linkage System that comprehensively links health and non-health data for the 2.5 million people living in Western Australia[28]. Regional linkage of administrative health data has also been undertaken in Canada, particularly Ontario[29] and British Columbia[30]. Furthermore, Nordic countries also collect administrative health data that can be linked at the individual level using a personal identifier that is common across all routine health datasets in the country of interest. The policy-focussed five-year CVD risk equations developed from 2006 VARIANZ cohort data, for instance, are being replicated by the Danish Heart Foundation and The University of Southern Denmark using linked Danish administrative data obtained from Statistics Denmark.

## Strengths and Limitations

### Strengths

The comprehensiveness of the VARIANZ data is one of their most notable strengths. For example, compared to the 2006 New Zealand Census Estimated Resident population aged 20 years or older, the equivalent VARIANZ 2006 dataset represents a ‘response rate’ of 85% with higher levels of concordance in age groups over 55 years where CVD risk management and CVD events are most common. Inclusion of every individual identified in national health databases is a particular advantage for research involving ethnic sub-populations, as achieving adequate study power for these relatively small population groups can be challenging in traditionally recruited cohorts[31, 32]. Similarly, participant recruitment and retention issues encountered with other population groups such as those with mental illness or in studies encompassing socioeconomic status are also likely to be improved by using VARIANZ data. Use of the VARIANZ datasets can also minimise numerator/denominator mismatch for CVD-related estimates in defined sub-populations such as ethnic groups[18]. Nevertheless, health contact cohorts will still be affected by some degree of selection bias (for example, the variable population capture by age and ethnic group already discussed) as well as attrition bias, confounding and mediation (for example, increased likelihood of hospitalisation among more deprived individuals due to reduced utilisation of primary health care and increased disease severity).

VARIANZ data have been collected during routine interactions with the health system across New Zealand rather than in selected health care settings such as a sample of primary care providers or hospitals, which aids the generalisability of the research findings. All data are anonymised prior to record linkage to maintain data privacy for included individuals. Use of routinely collected health data also reduces the potential for loss to follow-up, as CVD-related health service contacts are automatically recorded in the national datasets and individuals who move residence within New Zealand would remain in the dataset. However, people who move overseas cannot be identified.

Linkage to other routinely collected regional or national New Zealand health data that are not currently included in the dataset can be undertaken to enable studies investigating CVD comorbidities or novel CVD risk factors to be conducted. For example, within the health databases used to identify the health contact population, data are available for all non-CVD related hospitalisations. The VARIANZ datasets can also incorporate data at the individual level from other available health databases such as the New Zealand Cancer Registry and the Programme for the Integration of Mental Health Data (PRIMHD) that both capture national data, and laboratory test results that are available for selected regions within New Zealand. Consequently, the datasets are ideal for examining variation across demographic or clinically-defined groups in the use of hospital services, outpatient clinics and pharmacotherapy or other research questions relating to treatment, outcomes and prognosis for CVD and related conditions.

### Limitations

Other than the variable population capture by age and ethnic group that has already been discussed, the main limitations of the VARIANZ health contact datasets relate to issues of data availability and data quality.

Certain relevant data are not available in the New Zealand health databases. For example, some CVD risk factor data such as smoking status and blood pressure measurements are not routinely collected at a national level. Furthermore, national Census data (regarding smoking status) and self-reported data from national health survey samples (regarding smoking, blood pressure and other risk factor data) do not encompass the NHI personal identifier and are, therefore, unable to be linked to the VARIANZ datasets using the current linkage methodology. Diagnoses recorded in primary care or on discharge summaries from the private health sector are also not currently available nationally and, therefore, cannot be used to augment records of prior publicly-funded hospitalisations for CVD, heart failure and atrial fibrillation when identifying a history of these conditions using the VARIANZ datasets.

The impact of missing data from the private health sector and primary care records on identification of prior CVD is likely to be small for two reasons. In the first instance, most individuals who access privately-funded medical care do so for non-acute conditions and will have had interactions with the public health sector for almost all acute care. Therefore, any significant CVD-related conditions are likely to have been recorded in one of the national health databases, particularly given the long look-back period used to identify a history of cardiac-related hospitalisations (1993-2006 for the VARIANZ 2006 dataset). Secondly, data from the PREDICT cohort[33, 34] that captures around 90% of the population eligible for CVD risk assessment in the Auckland and Northland regions of New Zealand indicates that around 25% of individuals with CVD recorded nationally from any source are diagnosed solely in primary care[35]. However, prior CVD in the VARIANZ data is identified from diagnoses that are severe enough to necessitate hospitalisation, which represent the most definitive CVD diagnoses. The follow-up data on non-fatal CVD events (occurring between 2007 and 2011 for VARIANZ 2006 data) are also constructed using the national dataset of publicly funded hospitalisations. Hence, these variables are similarly affected by misclassification as non-fatal CVD events that have been solely managed in primary care, in the private sector or overseas would not appear in the New Zealand hospitalisation database. The impact of missing data for non-hospitalised comorbid conditions such as renal disease is likely to be greater than for identification of CVD history, with the exception of diabetes which is relatively comprehensively captured in the Virtual Diabetes Registry.

Misclassification error due to miscoding of hospitalisations [36-40] and cause of death[36, 38-40] will also affect the VARIANZ datasets, although mortality data tend to be coded more accurately[36, 38]. Furthermore, the national pharmaceutical databases only capture subsidised dispensing episodes from community pharmacists. While this encompasses the vast majority of dispensing activity, in-hospital medications and over-the-counter purchases (for example, of aspirin) would not appear in the national pharmaceutical dispensing database.

Finally, ethnicity coding in New Zealand health data until now has enabled identification of Indian individuals, but non-Indian South Asian peoples (who are also at high risk of CVD) have not been able to be differentiated from the Other Asian group. However, updated ethnicity data protocols recently released by the Ministry of Health should enable more granular identification of ethnic groups in the near future.

## Data Access

Researchers interested in using the VARIANZ datasets in collaboration with the VIEW programme should contact Professor Rod Jackson at rt.jackson@auckland.ac.nz. The VIEW research programme data access procedures incorporate the ‘Five Safes’ principles, an internationally recognised risk assessment framework encompassing safe projects, safe people, safe settings, safe data and safe output[41].

To ensure safe projects are conducted using the VARIANZ data, a template will be provided to allow completion of a data access proposal (DAP) that outlines the proposed research and the specific data required. The DAP will then be considered by the VIEW Leadership team, which is comprised of senior academics, including Professor Rod Jackson and Associate Professor Matire Harwood who are co-directors of the research programme. Links to the profiles of each of the Leadership team are available at: https://www.fmhs.auckland.ac.nz/en/soph/about/our-departments/epidemiology-and-biostatistics/research/view-study.html The DAP applications will be considered according to the consistency of the research proposal with VIEW programme objectives (listed on the website above), originality of the study question and robustness of the methodology. If the proposal involves analyses by ethnic group, then the DAP will also be considered by the VIEW Maori, Pacific and South Asian Governance Groups who provide ethnic oversight of all research conducted by the VIEW programme.

The research credentials of the applicant will also be reviewed. If approved, researchers are required to adhere to the VIEW team Code of Practice and to sign a Data Release Agreement form that outlines the conditions for data storage and use that must be adhered to (i.e. “safe people”).

In terms of safe settings, source data and research-ready VARIANZ data are not publicly accessible. Researchers will only be able to access data through a VIEW virtual machine that is separate from the wider University network, is disconnected from the internet and is not accessible by USB. Approved researchers are expected to be physically based at the Section of Epidemiology and Biostatistics at the University of Auckland for a few days at the outset of data access. Subsequently, remote access to data can be arranged. Access to source data is highly restricted and requires authorisation from the VIEW team data manager. These data access arrangements are not expected to change in the foreseeable future.

With consideration of the “safe data” principle, all data made available to researchers are anonymised; the VIEW team does not have access to identifiable health data and therefore the potential for re-identification of research-ready data is minimised. Finally, including a VIEW team member as a co-investigator for all approved research projects involving VIEW data contributes to safe outputs, as all results are reviewed by at least one VIEW team member for any identifying results such as small counts.

## Conclusion

The VARIANZ datasets contain detailed individual-level linked administrative health data regarding CVD and related conditions. The full datasets capture the majority of the New Zealand population in a given year from 2006 onwards. Subsets of the VARIANZ datasets can also be identified based on specific health contacts such as CVD hospitalisations only, and can be requested for years prior to 2006 subject to data availability. VARIANZ data for a given year can be individually-linked to data in subsequent years to create VARIANZ cohort studies. Bespoke linkage can also be undertaken to include other national and regional administrative health data such as non-CVD related hospitalisations to enable exploration of CVD comorbidities or novel risk factors. The VARIANZ datasets are a rich CVD data resource that can be used to explore a range of research questions regarding the management, outcomes and prognosis for CVD.

## Appendix 1: Overview of key quality checks undertaken by the New Zealand Ministry of Health for selected National Data Collections (National Collections and Reporting, New Zealand Ministry of Health, personal communication 2019)

Cross checking is undertaken by the Ministry of Health across national collections. For example, if someone dies, then checks are undertaken to confirm that reporting ceases across other collections. Where data inconsistencies are detected, for example with demographic data, the Ministry of Health follows up with General Practitioners and District Health Boards to clarify and amend details.

**1) National Health Index demographic data**

National Health Index (NHI) demographic data can only be updated by selected individuals who have had appropriate training, including hospital administrators, some Primary Health Organisation administrators, Ministry of Health contact centre staff and NHI analysts at the Ministry of Health. If an individual’s demographics are altered substantially, then the corresponding NHI number is included in a report to Ministry of Health analysts who investigate that record. All external organisations who connect to NHI data must first undergo compliance testing to ensure that no corruption of the NHI data occurs.

**2) Hospitalisation admissions**

In addition to District Health Board internal and inter-Board audits of health data, the Ministry of Health runs regular checks of data submitted to the Hospital admissions collection, involving all aspects of the records including clinical coding. The Ministry of Health then reports potential data errors back to District Health Board clinical coding units, who are required to review all flagged records, correct data errors, and re-submit the corrected records to the Hospital admissions collection. If common coding errors are detected, such as following a change to a coding standard as part of a classification upgrade, the Ministry also undertakes ad hoc data checks specific to the detected issue.

**3) Outpatient admissions**

Any organisation contributing data to the outpatient collection must first go through compliance testing. Regular quality reports are obtained looking for issues such as duplicate entries and feedback is subsequently provided back to the contributing District Health Board.

**4) Medication dispensing**

The pharmaceutical dispensing data can only be accessed by authorised staff from the Ministry of Health, the Pharmaceutical Management Agency of New Zealand and District Health Boards. The proportion of recorded dispensing episodes that can be linked to NHI numbers is regularly audited.

**5) PHO enrolment**

The PHO data is provided quarterly by PHOs to the Ministry of Health. Each record must have an NHI and the demographic data is compared to the data already held for that individual with clarification sought and amendment undertaken if required in the case of any significant differences.

**6) Mortality collection**

Data in the Mortality Collection is initially obtained from the Department of Internal Affairs death registrations. The Ministry of Health then adds information about the underlying cause of death from a wide range of sources depending on how the person died.

For example, data from Water Safety would be sought regarding a drowning death. Data from the Cancer registry and hospital records would be obtained for a death from cancer. The demographic details from the death-related data are compared with the NHI data to confirm the dead person’s identity. All death-related coding is reviewed by another clinical coder before the underlying cause of death is confirmed.
